# Shifts in Ectomycorrhizal Fungal Communities and Exploration Types Relate to the Environment and Fine-Root Traits Across Interior Douglas-Fir Forests of Western Canada

**DOI:** 10.3389/fpls.2019.00643

**Published:** 2019-05-22

**Authors:** Camille E. Defrenne, Timothy J. Philpott, Shannon H. A. Guichon, W. Jean Roach, Brian J. Pickles, Suzanne W. Simard

**Affiliations:** ^1^Department of Forest and Conservation Sciences, Faculty of Forestry, The University of British Columbia, Vancouver, BC, Canada; ^2^Ministry of Forests, Lands, Natural Resource Operations and Rural Development, Cariboo-Chilcotin Natural Resource District, Williams Lake, BC, Canada; ^3^Stable Isotope Facility, Department of Forest and Conservation Sciences, University of British Columbia, Vancouver, BC, Canada; ^4^Skyline Forestry Consultants Ltd., Kamloops, BC, Canada; ^5^School of Biological Sciences, University of Reading, Reading, United Kingdom

**Keywords:** biogeographic gradient, ectomycorrhizal fungi, exploration type, fine-root traits, forest ecosystems, functional ecology, mycorrhizas, *Pseudostuga menziesii* var. *glauca*

## Abstract

Large-scale studies that examine the responses of ectomycorrhizal fungi across biogeographic gradients are necessary to assess their role in mediating current and predicted future alterations in forest ecosystem processes. We assessed the extent of environmental filtering on interior Douglas-fir (*Pseudotsuga menziesii* var. *glauca* (Beissn.) Franco) ectomycorrhizal fungal communities across regional gradients in precipitation, temperature, and soil fertility in interior Douglas-fir dominated forests of western Canada. We also examined relationships between fine-root traits and mycorrhizal fungal exploration types by combining root and fungal trait measurements with next-generation sequencing. Temperature, precipitation, and soil C:N ratio affected fungal community dissimilarity and exploration type abundance but had no effect on α-diversity. Fungi with rhizomorphs (e.g., *Piloderma* sp.) or proteolytic abilities (e.g., *Cortinarius* sp.) dominated communities in warmer and less fertile environments. Ascomycetes (e.g., *Cenococcum geophilum*) or shorter distance explorers, which potentially cost the plant less C, were favored in colder/drier climates where soils were richer in total nitrogen. Environmental filtering of ectomycorrhizal fungal communities is potentially related to co-evolutionary history between Douglas-fir populations and fungal symbionts, suggesting success of interior Douglas-fir as climate changes may be dependent on maintaining strong associations with local communities of mycorrhizal fungi. No evidence for a link between root and fungal resource foraging strategies was found at the regional scale. This lack of evidence further supports the need for a mycorrhizal symbiosis framework that is independent of root trait frameworks, to aid in understanding belowground plant uptake strategies across environments.

## Introduction

Shifts in the taxonomic and functional structure of mycorrhizal communities across plant host distributions underpin changes in biogeochemical processes, such as modification of carbon (C) and nitrogen (N) cycles (Clemmensen et al., 2013; Koide et al., 2014; Cheeke et al., 2017; Wurzburger and Clemmensen, 2018; Jassey et al., 2018). Therefore, identifying the biotic and abiotic factors that shape mycorrhizal fungal communities is a prerequisite for understanding terrestrial ecosystem processes and predicting the impacts of global change on plant communities (Hazard and Johnson, 2018; Hoeksema et al., 2018; van der Linde et al., 2018). As climate changes, mycorrhizal fungi will likely respond to a range of environmental factors, and not necessarily the same factors as their hosts (Pickles et al., 2012), placing a premium on large-scale studies that examine communities across multiple environmental gradients (Lilleskov and Parrent, 2007; van der Linde et al., 2018).

Ectomycorrhizal fungi (EMF) play a dominant role in temperate and boreal forest ecosystems, where they control plant acquisition of soil resources (e.g., inorganic and organic forms of N and phosphorus, P; Read and Perez-Moreno, 2003; Heijden and Horton, 2009) and soil C dynamics (Simard and Austin, 2010). Biogeographic patterns in EMF diversity are now being studied (Tedersoo, 2017; Hu et al., 2019). However, there is a lack of baseline information on patterns of EMF community composition and functional trait distribution, especially at large spatial scales such as the regional (i.e., scale of a country), continental or global scales (Tedersoo, 2017; van der Linde et al., 2018; Hu et al., 2019). Yet, biogeographic data on EMF community structure are necessary to assess their role in mediating current and predicted alterations in the C cycle (Jassey et al., 2018), the hydrologic cycle (Bjorkman et al., 2018) or plant productivity (Coops et al., 2010; Richardson et al., 2018).

At the continental scale, patterns of EMF community composition of *Pinus sylvestris*, *Picea abies*, and *Fagus sylvatica* have been investigated in Europe, where host plant family and N deposition had the predominant filtering effects (Tedersoo et al., 2012; Põlme et al., 2013; Suz et al., 2014; Rosinger et al., 2018; van der Linde et al., 2018). Within Europe, at the regional scale, N deposition, rainfall and soil moisture were also found to drive shifts in the *P. sylvestris* EMF community structure (Jarvis et al., 2013), whereas other European studies highlighted the filtering effects of temperature and soil fertility on EMF communities (Sterkenburg et al., 2015; Pena et al., 2017). In Western North America, Pickles et al. (2015a) have also inferred from a common-garden greenhouse study on interior Douglas-fir (*Pseudotsuga menziesii* var. *glauca* (Beissn.) Franco; hereafter Douglas-fir) seedlings that temperature and soil fertility may drive habitat filtering in EMF communities.

Across environments, variation in EMF functional traits may relate better to ecosystem processes than variation in EMF species composition because it informs how groups of species function and the extent that there is functional redundancy in species diversity (Koide et al., 2014; Hazard and Johnson, 2018). For instance, EMF functional traits such as enzymatic activity (Courty et al., 2016), N preference (Leberecht et al., 2015; Haas et al., 2018), mycelial hydrophobicity or the differentiation of extraradical hyphae (i.e., exploration type; Agerer, 2001, 2006; Jarvis et al., 2013; Pickles et al., 2015a; Fernandez et al., 2017; Ostonen et al., 2017; Pena et al., 2017; Köhler et al., 2018; Rosinger et al., 2018) have been shown to impact ecosystem processes (Koide et al., 2014). Exploration type is a functional trait that connects the morphology and differentiation of EMF hyphae to differences in nutrient acquisition strategies. From a functional perspective, exploration type determines the ability of EMF to colonize new roots, form common mycorrhizal networks, or forage, acquire and transport resources (Agerer, 2006). Fungi with contact, short- and medium-distance smooth exploration types, for example, may preferentially use soluble, inorganic N forms (Lilleskov et al., 2002; Hobbie and Agerer, 2010). Alternatively, long-distance explorers may be more effective in capturing patchily distributed organic N (Koide et al., 2014), and are more likely to be resistant to decay due to their hydrophobicity. Hence, EMF fungi of the long-distance exploration type may drive soil C storage and C:N ratio (Suz et al., 2014), although some short-range EMF, including *Cenococcum geophilum* and *Cadophora Finlandia*, are also resistant to decay (Agerer, 2006; Fernandez et al., 2016).

Shifts in EMF exploration type may compensate for changes in fine-root structure. For example, across 13 temperate tree species, the abundance of larger absorptive fine roots, whose large diameter and associated high construction costs limits efficient resource foraging, was positively correlated with the proportion of longer distance exploration types, thus resulting in functional complementarity between fine roots and EMF with respect to soil resource capture (Chen et al., 2018a,b). This is because plants with coarser roots are less able to forage for and absorb soil resources, thus they should benefit the most from medium- or long-distance explorers that can acquire and transport resources well beyond root depletion zones (Chen et al., 2018a,b). To our knowledge, only three studies have linked root and EMF functional traits (Ostonen et al., 2011; Cheng et al., 2016; Chen et al., 2018a). Yet, studies connecting commonly measured economic fine-root traits (e.g., morphological, chemical and architectural traits) and mycorrhizal functional traits are essential for broadening root trait frameworks (McCormack et al., 2017).

Root density is also an important factor to consider when linking fine-root traits and exploration types because exploration type assemblage may be well predicted by root spacing (Peay et al., 2011) and conversely, EMF species influence root density (Pickles et al., 2010). This is especially important when working at the scale of meters or in primary succession settings (Peay et al., 2011). In this study however, we focused on economic traits commonly measured at the individual fine-root level.

To assess the extent of abiotic environmental filtering on EMF community taxonomic and functional structure, and to examine the relationship between fine root and EMF exploration type, we investigated patterns of belowground trait variation across five regions that differed in precipitation, temperature and soil fertility (pH, cation exchange capacity (CEC), total N and available P) in an area of *c*. 25, 300 km^2^ (49.6 to 51.7°N) in British Columbia, Canada. We focused on Douglas-fir in interior Douglas-fir dominated forests which are widely distributed from the Rocky Mountains of Canada and the United States to the mountains of central Mexico (Lavender and Hermann, 2014). Defrenne et al. (unpublished) have explored the variation in morphological, chemical and architectural traits among fine roots across the same biogeographic gradient and revealed that Douglas-fir trees from colder/drier climates had fine roots with higher diameter, lower root tissue density (RTD), and lower C:N, compared to trees from milder climates. In this study, we first hypothesized that temperature and soil fertility would be the main drivers of EMF diversity and community composition. Our second hypothesis was that medium-distance fringe or long-distance explorers would be more abundant in colder climates (see the third hypothesis) or in soils with high C:N ratio, because fungi with rhizomorphs that preferentially use insoluble, organic N may be more competitive for plant nutrition under these conditions. Building on the results of Defrenne et al. (unpublished), our third hypothesis was that EMF traits compensate for changes in fine-root structure, and especially root diameter, where colder/drier climates with larger diameter roots are dominated by EMF with medium-distance fringe or long-distance exploration types.

**Table 1:** Climatic and edaphic properties of the 15 study stands selected across a biogeographic gradient in Western Canada.

		Location			Climatic properties (1981–2010)		Soil properties			
Region	Site within region	Latitude (°N)	Longitude (°W)	Elevation (m)	MAT (°C)^a^	MAP (mm)^b^	Soil avail. P (ppm)	CEC (cmol(+)kg^−1^)^c^	Soil C:N	pH
Williams Lake	WL1	51.73	123.01	1149	3.1	485	25.7 ± 8.6	11.3 ± 2.4	23.2	6.5
	WL2	51.74	122.78	1087	3.5	477	59.2 ± 24.6	15.6 ± 4.6	21.7	6.5
	WL3	51.74	122.76	1080	3.6	476	62.9 ± 9.3	21.2 ± 5.3	20.4	6.8
Revelstoke	R1	50.80	118.00	762	5.3	1216	117.5 ± 68.4	4.8 ± 1.7	24.4	5.8
	R2	50.79	117.98	706	5.6	1212	33.8 ± 9.8	4.1 ± 3.0	33.5	6.0
	R3	50.83	118.02	726	5.7	1167	232.4 ± 79.5	8.3 ± 2.0	30.1	5.6
Kamloops	K1	50.89	120.33	945	5.4	448	81.2 ± 18.3	21.5 ± 14.2	18.5	6.6
	K2	50.92	120.28	895	5.8	434	47.8 ± 17.0	21.9 ± 7.3	17.3	6.4
	K3	50.92	120.28	939	5.6	442	46.4 ± 19.3	15.5 ± 4.3	17.8	5.9
Salmon Arm	SA1	50.76	119.19	712	6.4	688	3.6 ± 1.5	34.8 ± 11	32.4	7.6
	SA2	50.65	119.05	721	6.1	701	161.8 ± 41.5	10.8 ± 6.1	28.5	6.5
	SA3	50.79	119.06	703	6.3	696	225.6 ± 95.3	12.3 ± 5.7	33.9	6.6
Nelson	N1	49.55	117.72	671	7.0	886	124.1 ± 34.1	3.8 ± 2.3	34.3	5.9
	N2	49.60	117.75	679	7.5	860	328.9 ± 77.8	5.7 ± 3.1	28.2	5.9
	N3	49.61	117.77	754	7.3	856	379.3 ± 22.1	5.9 ± 2.5	27.0	6.1

*Soil properties are reported as mean ± standard error (SE, n = 5). ^a^MAT, mean annual temperature; ^b^MAP, mean annual precipitation; ^c^CEC, effective cation exchange capacity.*

## Materials and Methods

### Biogeographic Gradient

**FIGURE 1:**
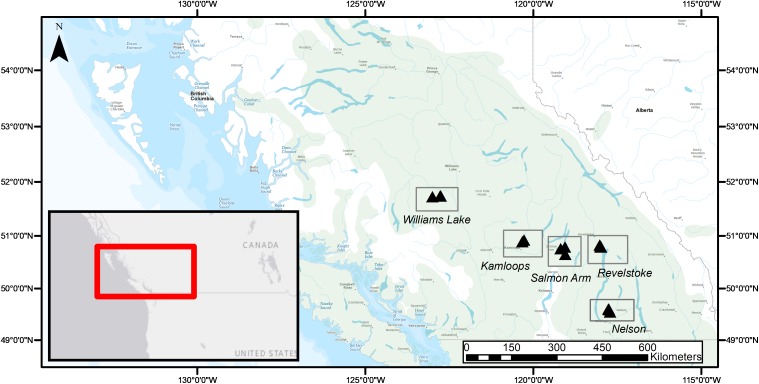
Geographical distribution of study regions (rectangles) and forest stands (3 triangles per study region) across the current natural range of interior Douglas-fir (*Pseudotsuga menziesii* var. *glauca*; green shading) in British Columbia, Canada.

Fine roots and EMF root tips were collected from five regions in three naturally regenerated, mature, closed-canopy forest stands (30 × 30 m) per region in summer 2016 (Figure 1). We selected the five regions to obtain a biogeographic gradient with substantial precipitation and temperature ranges (Table 1). Mean annual temperature (MAT) ranged from 3.4 to 7.3°C and was lowest in Williams Lake, followed by Revelstoke, Kamloops, Salmon Arm and Nelson. The driest region was Kamloops (average mean annual precipitation- MAP, 441 mm) and the wettest was Revelstoke (average MAP 1200 mm). We picked stands that were at least 400 m apart and ecosystems that best reflected the regional climate (namely, zonal site series; Meidinger and Pojar, 1991). Average stand age ranged from 98 years (Revelstoke) to 143 years (Salmon Arm), and the proportion of Douglas-fir by basal area ranged from 49% in the mixed, even-aged forest stands of Salmon Arm to 100% in the pure, uneven-aged forest stands of Kamloops. For the basal area estimates, all the trees with a DBH > 10 cm were measured (this included only mature trees; for further details on site and stand characteristics, see Supplementary Table 1).

The southernmost stands (Nelson, Revelstoke and Salmon Arm) occurred predominantly on Brunisolic soils that were characterized by lower CEC, soil pH and soil N compared to the northern most stands (in Williams Lake and Kamloops) which occurred on Luvisolic soils (British Columbia Ministry of Forests and Range and British Columbia Ministry of Environment, 2010). Climatic variables for the period 1981–2010 were obtained from ClimateNA (Wang et al., 2016) and soil samples were analyzed for total soil carbon (C) and N concentrations, available phosphorus (PO_4_–P; orthophosphate as phosphorus) and CEC (for further details on soil sample collection, see the next section and for details on soil sample analyses, see Supplementary Text 1A).

### Fine Root and Ectomycorrhiza Sampling and Processing

In each stand, single soil blocks (20 × 20 × 20 cm) were extracted from five Douglas-fir trees (200 cm from the trunk) in a manner that avoided clumping of sampling location (i.e., trees were at least 5 m apart). The soil blocks encompassed organic layers (L, F and H) and mineral horizons (A and B) to obtain a more complete vertical representation of the EMF community (Rosling et al., 2003). In addition, one organic (L, F, H layers) and one mineral soil sample (upper mineral horizons A and B, from the bottom of the organic layer to 10 cm depth) were collected using a trowel from each target tree (5 trees) to evaluate relationships between EMF communities and soil properties. A total of 75 sample sets were collected (5 regions, 3 stands per region, 5 trees per stand) and stored at 4°C until processing (up to 3 months).

To recover Douglas-fir fine roots and ectomycorrhizas, each soil block was soaked in water overnight before being washed over a 4 mm screen. All fine-root branches (<2 mm diameter) and fragments (>3 cm length) were collected from the sieve and sorted by tree species (based on the morphological key described in Supplementary Table 2). To guarantee random selection of EMF root tips, root fragments from each soil block were laid out on a numbered grid, and grid cells were selected using a random number generator. We examined and cleaned root fragments with a soft brush under the microscope until *c.* 50 live fine-root tips/soil block were collected. Excised fine-root tips were classified as individual morphotypes (based on the presence of a fungal mantle and according to Goodman et al., 1996) or uncolonized (root hairs present, or no visible mantle, usually unbranched). All tips were frozen at –80°C but only 5–10 tips per morphotype, across all soil blocks for the entire study, were used for later DNA analysis.

To assess the effect of fine-root traits on EMF taxonomic and functional diversity (exploration type), a total of 365 Douglas-fir fine-root branches was divided into individual root orders following the morphometric classification approach of Pregitzer et al. (2002). In this classification, the most distal, unbranched roots are first order and second-order roots begin at the junction of two first order roots, and so on. First-order roots were either colonized by EMF or were unbranched and the root tips uncolonized. We avoided thicker, longer pioneer first-order roots (Zadworny and Eissenstat, 2011). Each first-order group (i.e., all first-order roots of a given branch) was scanned separately and analyzed for morphological features using WinRHIZO (total length, total surface area, average diameter and total volume; 400 dpi, 165 -level gray scale, EPSON Perfection V800 Photo, STD 4800; WinRHIZO pro 2016 software, Regent Instruments Inc., Quebec City, Canada). For each first-order group, we determined specific root length, SRL (m g^−1^), specific root area, SRA (cm^2^ g^−1^), and RTD (mg cm^−3^). In addition, a subsample of 180 first-order roots were randomly selected and analyzed for C and N concentration (%) (see Supplementary Text 1B). These traits were selected for analysis due to their expected relationships with plant investment into root construction and maintenance and their benefits for soil resource foraging and acquisition.

### Molecular Analyses of Ectomycorrhizas

Five to ten frozen EMF root tips for each of the 97 putative morphotypes (across all soil blocks for the entire study) were pooled and ground in liquid nitrogen before extracting fungal DNA using DNeasy^®^ PowerSoil^®^ kit, according to the manufacturer instructions (Qiagen^®^, 2017, ON, Canada). Fungal DNA extracts were sent to the Centre for Comparative Genomics and Evolutionary Bioinformatics (CGEB) at Dalhousie University, Halifax, Canada. High-throughput sequencing (Illumina MiSeq v3 chemistry, 600 cycles; Illumina, San Diego, CA, United States) was used to identify the target EMF OTU. For the library preparation, amplicon fragments were PCR-amplified with fungal specific primers (ITS86F, GTGAATCATCGAATCTTTGAA; ITS4R, TCCTCCGCTTATTGATATGC) targeting the internal transcribed spacer region 2 (ITS2) (variable length, avg. *c*. 350 bp) of ribosomal DNA (White et al., 1990; Turenne et al., 1999; Vancov and Keen, 2009). Primers contained Illumina barcodes and overhang adaptors, allowing for a one-step PCR preparation of sequence libraries.

DNA sequencing results were analyzed using the QIIME2^TM^ bioinformatics platform (Caporaso et al., 2010). The software package DADA2 was used to assemble bidirectional reads while filtering for quality and dereplicating sequences (Callahan et al., 2016). Prior to taxonomic assignment, representative sequences were exported from QIIME2 into fasta format and then ITS2 regions were extracted, chimeras were detected and non-ITS2 sequences were screened out using the software tool ITSx (Bengtsson-Palme et al., 2013). Extracted ITS2 sequence data were imported back into QIIME2 and the corresponding QIIME2 feature table was filtered to remove non-ITS sequence data. Demultiplexed, quality-controlled ITS2 sequence data were further screened for chimeras and then clustered into operational taxonomic units (OTUs) at 99% sequence similarity using a *de novo* clustering method with VSEARCH (Westcott and Schloss, 2015; Rognes et al., 2016).

For fungal species identification, we used the Basic Local Alignment Search Tool (BLAST) against the National Center for Biotechnology Information (NCBI) GenBank and UNITe public sequence databases (Abarenkov et al., 2010). We used two criteria to assign species or genus names to each morphotype: (i) Only EMF OTUs were considered (no consideration of root-associated fungi such as saprotrophs, root endophytes, molds or pathogens), and (ii) where pairwise identity (i.e., the amount of nucleotide that matches exactly between two different sequences) corresponding to the indicated EMF species was higher than 97%. In addition, morphotype characteristics were compared to reference photos from the Ectomycorrhizae Descriptions Database^1^ and the DEEMY database ^2^. Using this method, 82% of the morphotypes were identified to the species or genus level. For all but ten morphotypes, the assigned EMF species or genus corresponded to the EMF OTU with the highest number of reads; for the remaining ten morphotypes (morphotype IDs 81, 72c, 50, 55, 49, 34b, 31, 25, 3 and 2, see Supplementary Table 3), the EMF OTU with the highest number of reads had a low pairwise identity (<94) or was likely a contaminant. In this case, the EMF OTU with the second or third highest number of reads was chosen, but only if the morphology of the morphotype corresponded to the photos from the databases.

For each species or genus, exploration type (contact, short-distance, medium-distance smooth, medium-distance fringe and long-distance) was assigned after Agerer (2001, 2006) and compared to the published data from Ostonen et al. (2017), Pena et al. (2017), and Fernandez et al. (2017). For the genus *Sistotrema*, we referred to Ostonen et al. (2017) because it was not included in Agerer (2001, 2006). We assumed that exploration type was conserved within a genus as we did not study EMF genotypic trait variation. This assumption has been made in many other studies although plasticity of EMF mycelium can be substantial and should be taken into account more consistently (Hazard and Johnson, 2018; van der Linde et al., 2018). Hydrophobicity was assigned based on Lilleskov et al. (2011) and Fernandez et al. (2017).

### Data Analyses

All statistical analyses were conducted in R version 3.5.1 (R Core Team, 2018) and results were considered statistically significant at *P* < 0.05. Fungal richness was examined across regions by estimating components of α-diversity: (i) observed species richness in each soil block and (ii) richness estimators: Chao1 (Chao, 1984), first- and second-order Jackknife (Burnham and Overton, 1979). Diversity patterns were examined by calculating the following diversity indices: Shannon–Wiener Diversity Index (*H*′ = − ∑ *pi* ln *pi*), Shannon’s Evenness (E), and Simpson’s Index of Diversity (1 − *D*; *D* = 1 − ∑ *pi*^2^), where *pi* is the proportion of species *i* relative to the total number of species in a sample. We assessed the effect of region on EMF richness, evenness and diversity using a nested ANOVA (linear mixed effect model) with region as a fixed effect and site nested within region as a random effect using the function “*lmer*” from the *lme4* package. We did not rarefy to the lowest sampling depth (i.e., we only collected 12 root tips in a soil block from Revelstoke) because rarefaction curves indicated that our sampling effort was sufficient as it resulted in EMF species saturation (Supplementary Figure 1).

To investigate the effects of environment and fine-root traits on the taxonomic and functional structure of EMF communities, we first calculated the variance inflation factor (VIF) for each environmental (MAT, MAP, soil C:N, CEC, pH, soil available phosphorus) and root trait (SRL, SRA, diameter, RTD, root C:N) predictor, to avoid multicollinearity among predictive variables, using the “*vif*” function from the *usdm* package (Naimi et al., 2014). Predictors with the highest VIF were sequentially dropped until all VIF values were below three (Zuur et al., 2010). This process removed CEC and soil N from the environmental predictors and SRL from the root trait predictors. Second, unidentified morphotypes (from which DNA was not extracted and for which a sequence was not found) were removed before all analyses on the basis that reconsideration of photographic evidence suggested that they were most likely to have been dead root tips. A Hellinger transformation was applied to species and exploration type data matrices. We used a distance-based redundancy analysis (db-RDA; Legendre and Anderson, 1999) to examine β-diversity based on Bray–Curtis dissimilarities using the “*capscale*” function in *vegan.* The best model was chosen utilizing forward model selection with permutation tests (*P*-value for variable retention = 0.05). The general form of the models was:

#### EMF Species or Exploration Type – Environmental Factors (e.g., MAT, MAP) or Root Traits (e.g., SRA, RTD)

Models were tested for significance using permutational multivariate analysis of variance (PERMANOVA, function “*adonis*” in *vegan*, 999 permutations), after assessing the multivariate homogeneity of regions dispersions (function “*betadisper*” in *vegan*; Oksanen et al., 2018; Supplementary Figure 2). Significant PERMANOVA effects were assessed using *post hoc* pairwise contrasts (function “*multiconstrained*” from the package *BiodiversityR*; Kindt, 2018).

The db-RDA /PERMANOVA approach was not developed to account for nested data (here, sites are nested within regions). Thus, to complement these analyses, we used a two-way approach, that only worked with two nested factors: (i) For the effect of sites within regions, we used PERMANOVA with permutations constrained within sites (strata = site in “*adonis*”) and (ii) for the effect of regions, we ran a nested analysis of variance with the function “*nested.npmanova*” from the package *BiodiversityR*. These two complementary analyses were run on the models:

#### EMF Species or EMF Exploration Types – Regions/Sites

In addition, to account for the presence of mean-variance relationships in multivariate community analyses, we built multivariate generalized linear models using the package “*MVABUND*” (Wang et al., 2012). An offset (log of row sums) was added to the models to standardize the response variables and account for the unequal sample size. Models were run twice: with all the species and without the unresponsive species (coefficient < |5| ). Model significance was tested with a likelihood-ratio test and univariate *P*-values were adjusted for multiple testing using a step-down resampling procedure.

## Results

### Identification and Taxonomic Diversity

In this study, 3914 fine-root tips were extracted from 75 soil blocks and sorted into 97 putative EMF morphotypes (on average, 4.0 ± 0.2 morphotypes per soil block) of which 82 EMF morphotypes were successfully sequenced. The sequencing of the 82 morphotypes resulted in 6,322,065 sequences that were clustered into 1,901 OTUs. These OTUs comprised the following guilds: EMF, saprotrophs, root endophytes, molds or pathogens. Considering all the guilds, the average number of OTUs per morphotype was 69.0 ± 3.6 (Supplementary Table 3). Out of these 69 fungal OTUs per morphotype, on average, 10 OTUs were EMF (highlighted in red in Supplementary Table 3). After using the criteria described in the section “Molecular analyses of ectomycorrhizas,” we assigned a unique EMF OTU to each of the 82 morphotypes. We then obtained 54 unique EMF OTUs because some morphotypes were assigned the same OTU (Supplementary Table 4). Of these 54 EMF OTU, 46 and 54% were identified to genus and species, respectively. Of the 54 EMF taxa, 91% were Basidiomycota and 9% were Ascomycota. In addition, 33% were resupinate fungi, 13% were hypogeous and 54% were mushroom-forming fungi. Tips from the 15 EMF morphotypes for which no sequences were obtained (most likely representing dead root tips) represented 10% of all the mycorrhizal root tips.

Species accumulation curves showed that almost the entirety of species richness was recovered for Kamloops. Alternatively, we recovered *c.* 80% of the estimated species richness for the remaining regions (Supplementary Figure 1). Richness estimators (Chao1, Jack1, Jack2) were similar to the observed species richness, which confirm that only a small number of species were not accounted for with our sampling scheme (Supplementary Table 5A). Revelstoke had the highest species richness per region with 25 species compared to 24 species in Salmon Arm and Williams Lake, followed by Nelson with 23 species and Kamloops with 20 species. We did not detect any differences in α-diversity among the five regions, where species richness averaged four EMF species/soil block for each region (Supplementary Table 5B). Similarly, species evenness and diversity estimated with Shannon and Simpson’s diversity indexes were low, averaging 0.8 and 0.5, respectively, and did not vary by region.

### Taxonomic Composition

**FIGURE 2:**
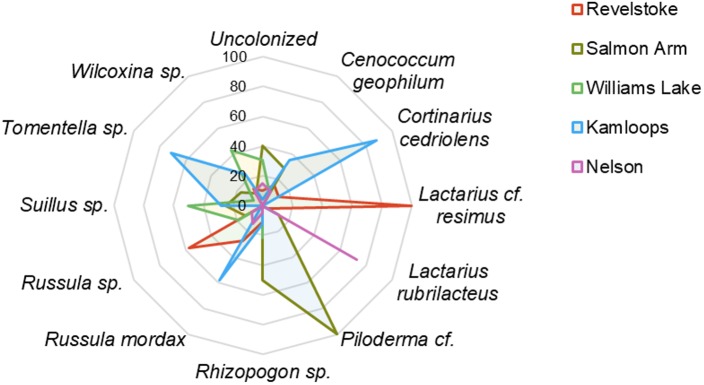
Relative abundance (%) of ectomycorrhizal taxa on interior Douglas-fir among five regions in western Canada. Only the species/genera representing >2% of root tip abundance were included. For a given ectomycorrhizal species, numbers represent the percentage of root tips colonized by this species in each region.

Across the environmental gradients, the most abundant OTUs identified at the species level with >2% root tip abundance were *C. geophilum* (8.3%), *Lactarius rubrilacteus* (8%), *Russula mordax* (4%), *Lactarius* cf. *resimus* (2.5%), and *Cortinarius cedriolens* (2.1%), and the most abundant OTUs identified at the genus-only level were *Russula* sp. (8.4%), *Rhizopogon* sp. (5.6%), *Piloderma* cf. (3.3%), *Tomentella* sp. (3.3%), *Wilcoxina* sp. (3.4%) and *Suillus* sp. (2.5%; Figure 2; Supplementary Table 4). The five regions had five species/genera in common: *C. geophilum, Rhizopogon* sp., *Wilcoxina* sp*., Piloderma* sp. and *Russula* sp. The vast majority of fine-root tips colonized by *Russula* sp. and *Lactarius resimus* were found in the wettest region (Revelstoke; Figure 2), whereas the majority of root tips colonized by *C. geophilum*, *Tomentella* sp., *Russula mordax* and *Cortinarius cedriolens* were found in the driest region (Kamloops). Almost half (43%) of the occurrence of *Wilcoxina* sp. and half of the occurrence of *Suillus* sp. were in the coldest region (Williams Lake). Alternatively, 24 and 11% of the occurrence of *Wilcoxina* sp. were in the warmest regions of Kamloops and Nelson, respectively, while the other half of *Suillus* sp. was also in warm regions (Salmon Arm and Kamloops). 75% of the occurrence of *Lactarius rubrilactus* was in the warmest region (Nelson).

**Table 2:** Effect of climatic and edaphic conditions on interior Douglas-fir ectomycorrhizal fungal species community composition across five regions assessed by **(A)** PERMANOVA and **(B)** assessed by multivariate generalized linear model. Effect of climatic and edaphic conditions on **(C)** exploration types assessed by PERMANOVA.

(A) Ectomycorrhizal fungal community composition, PERMANOVA model				
Abiotic factors	*Df*	*Pseudo F*	*Pseudo R^2^*	*P*-value
MAP^a^	1	2.38	0.03	**<0.01**
MAT^b^	1	2.94	0.04	**<0.01**
soil C:N^c^	1	2.48	0.03	**<0.01**
pH	1	1.79	0.02	**0.02**
Residuals	69		0.88	
Total	73		1.00	

**(B) Ectomycorrhizal fungal community composition, multivariategeneralized model**				

**Abiotic factors**	***Res.Df***	***Df.diff***	***Deviance***	***P*-value**

(Intercept)	61			
MAP	59	1	121.37	**0.01**
MAT	60	1	61.90	**0.01**
Soil C:N	58	1	98.22	**0.04**
pH	57	1	102.44	0.05

**(C) Ectomycorrhizal fungal exploration type, PERMANOVA model**				

**Abiotic factors**	**Df**	**Pseudo F**	***Pseudo R^2^***	***P*-value**

MAP	1	5.4	0.07	**<0.01**
MAT	1	4.5	0.05	**<0.01**
soil C:N	1	3.94	0.05	**0.01**
Residuals	69		0.83	
Total	72		1.00	

*Significant P-values (<0.05) are shown in bold. A negative binomial distribution was assumed for the multivariate model and explained deviance was tested after 999 permutations. ^a^MAP, mean annual precipitation; ^b^MAT, mean annual temperature; ^c^soil C:N, soil carbon-to-nitrogen ratio.*

**FIGURE 3:**
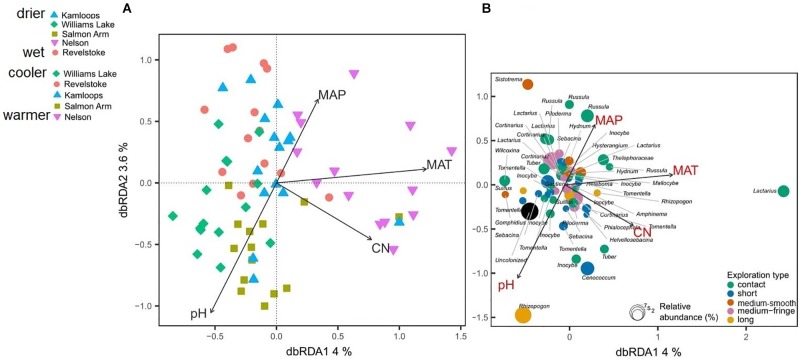
Distance-based redundancy analysis (db-RDA) sample **(A)** and species **(B)** ordinations based on ectomycorrhizal fungal abundance on interior Douglas-fir roots across five regions in western Canada. Only the variation explained by environmental variables is visualized. The ectomycorrhizal species are color-coded by fungal exploration type and are sized according to their relative abundance. The species epithet (when known) was removed to improve readability. MAP, mean annual precipitation; MAT, mean annual temperature; CN, soil carbon-to-nitrogen ratio. To aid comparison, predictor variables were standardized (*z*-scores) prior to analysis and are therefore unitless. Contact-, short- and medium-distance smooth exploration types are hydrophylic while medium-distance fringe and long-distance exploration types are hydrophobic.

Differences in climatic and edaphic conditions among the regions explained 12.2% (adjusted *R*^2^ = 0.07) of the variation in the Douglas-fir EMF community composition (Table 2A). The first axis of the db-RDA was mostly explained by differences in MAT (score = –0.89) and soil C:N ratio (score = –0.55) and separated the EMF communities of the warmest region (Nelson, MAT = 7.3°C) from those in colder regions (William’s Lake, MAT = 3.4°C; Kamloops, *c.* 5.6°C; Revelstoke, MAT = 5.5°C; Figure 3A). The second axis of the analysis was best explained by the gradients in precipitation (score = 0.53) and soil acidity (score = –0.79) and separated fungal communities in drier (Williams lake) and weakly calcareous soils (Salmon Arm) from those in wetter, strongly acidic Brunisolic soils (Revelstoke).

Genera such as *Tomentella, Cenococcum* and *Sebacina*, classified as the short-distance exploration type, were commonly associated with low MAT, soils richer in N and mid to low MAP (Figure 3B) and more generally, the short-distance exploration type taxa clustered in colder/drier regions. Fungal species such as *Rhizopogon* sp. and *Suillus* sp. with the long-distance type were exclusively found in drier climates while medium and medium-fringe explorers such as *Hydnum* sp., *Cortinarius* sp. and *Piloderma* sp. clustered in wet climates. Contact explorers such as *Russula* sp. and *Lactarius* sp. had a broader environmental range compared to the other exploration types but tended to be more abundant in wet climates. Uncolonized root tips occurred exclusively in regions with low MAT, low MAP and rich soils (high total N). Sites nested within regions did not have a significant effect on the EMF community composition, as revealed by the PERMANOVA with permutation restricted within sites (Supplementary Table 6A). However, the nested PERMANOVA confirmed the significant effect of regions (*P*-value = 0.001; Supplementary Table 6B)

**FIGURE 4:**
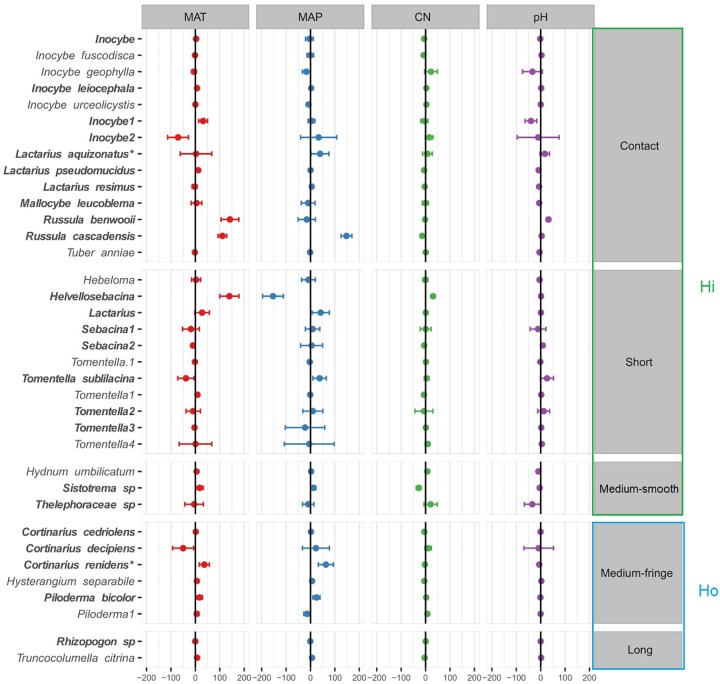
Ectomycorrhizal fungal species-specific response to environmental factors based on multivariate generalized linear models. Only species that were responsive were added to the model (coefficient > |5| ). Circles (•) represent species coefficients and lines, 95% confidence intervals. Species were grouped by exploration type indicated at the right-hand side of the plot, MAT, mean annual temperature; MAP, mean annual precipitation; CN, soil carbon-to-nitrogen ratio. To aid comparison, predictor variables were standardized (*z*-scores) prior to analysis and are therefore unitless. Hi, hydrophylic; Ho hydrophobic.

Species-specific responses to the environmental gradients were obtained using multivariate generalized linear models (Figure 4; only the most responsive species, with a coefficient > |5| were included in this model, see Supplementary Figure 3 for results with all the species). In agreement with the PERMANOVA model (except for soil pH), MAT, MAP and soil C:N ratio were all significantly related to shifts in EMF species across regions (Table 2B), yet EMF species were more responsive to MAP and MAT than soil C:N ratio (larger effect size; Figure 4). Generally, most taxa in the Russulaceae responded in a similar fashion, with species increasing in abundance with MAT and MAP (except for *Russula benwooii*). Response to climate was not similar in the Cortinariaceae and Sebacinaceae. For instance, *Cortinarius renidens* and *C. decipiens* expressed opposite responses to MAT, positive and negative, respectively, but had matching responses to MAP. Similarly, the genera *Sebacina* responded moderately but positively to MAP and negatively to MAT which was opposite to the response of the related genus *Helvellosebacina.*

### Exploration Types

**FIGURE 5:**
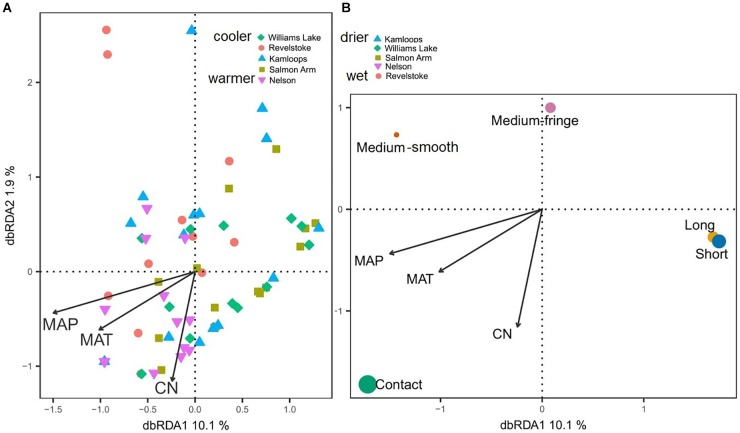
Distance-based redundancy analysis (db-RDA) sample **(A)** and exploration type **(B)** ordinations based on ectomycorrhizal fungal exploration type on interior Douglas-fir across five regions in western Canada. The ectomycorrhizal fungal exploration types are sized according to their relative abundance. MAP, mean annual precipitation; MAT, mean annual temperature; CN, soil carbon-to-nitrogen ratio. To aid comparison, predictor variables were standardized (*z*-scores) prior to analysis and are therefore unitless. Contact-, short- and medium-distance smooth exploration types are hydrophylic while medium-distance fringe and long-distance exploration types are hydrophobic.

Considering all morphotypes, 36.4% of mycorrhizal root tips were contact-distance type, 25.5% were short-distance type, 20.0% medium-distance fringe type, 7.3%, medium-distance smooth type, and 7.3% long-distance type (Supplementary Figure 4 and Supplementary Table 4). Precipitation and temperature explained 14% (adjusted *R*^2^ = 0.14; Table 2C) of the variation in the dominant exploration types across the gradient. Only the first axis of the db-RDA was significant and represented the variation in MAP (score = –0.83; Figure 5A) and to a lesser extent MAT (score = –0.56). This axis separated long- and short-distance exploration types occurring in drier/colder regions from contact and medium-distance smooth exploration types in wetter regions (Figure 5B). We found no significant effect of site on exploration type abundance but found an effect of region (*P*-value = 0.02; result not shown), whereas multivariate generalized linear models did not yield significant results (result not shown).

### Fine-Root Traits and Fungi

**FIGURE 6:**
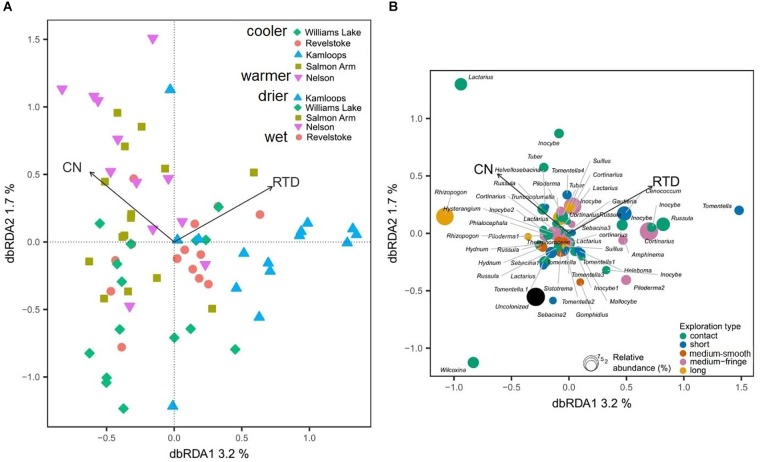
Distance-based redundancy analysis (db-RDA) sample **(A)** and species **(B)** ordinations based on ectomycorrhizal fungal abundance on interior Douglas-fir across five regions. Only variation explained by Douglas-fir fine-root traits is visualized. The ectomycorrhizal species are color-coded by fungal exploration type and are sized according to their relative abundance. The species epithet (when known) was removed to improve readability. CN, fine-root carbon-to-nitrogen ratio; RTD, fine-root tissue density. To aid comparison, predictor variables were standardized (*z*-scores) prior to analysis and are therefore unitless. Contact-, short- and medium-distance smooth exploration types are hydrophylic while medium-distance fringe and long-distance exploration types are hydrophobic.

Douglas-fir fine-root morphological and chemical traits explained 5% (adjusted *R*^2^ = 0.02; Table 3) of shifts in EMF species community structure. Only the first axis of the db-RDA was significant and was represented by the variation in first-order root C:N ratio (score = –0.72) and RTD (score = 0.83; Figure 6A). This axis separated the symbionts associated with fine roots of low RTD (in Williams Lake and Nelson) from those associated with fine roots of low C:N ratio (mainly in Kamloops). Fungal taxa such as *Wilcoxina*, *Tomentella* and *Sebacina* that were classified as contact and short exploration types tended to cluster together and were related to fine roots with low RTD (Figure 6B). Similarly, uncolonized root tips were all associated with fine roots of low RTD. Alternatively, medium-fringe explorers such as *Cortinarius*, *Piloderma* and *Amphinema*, as well as the short distance explorer, *Cenococcum* tended to be more abundant on fine roots with high RTD. The multivariate generalized linear model did not yield significant results (result not shown).

## Discussion

The wide gradient in climate and soil fertility across southern British Columbia was ideal for investigating the extent of environmental filtering on EMF community taxonomic and functional structure (exploration type) across populations of Douglas-fir. Our first hypothesis was partly rejected because climate and soil fertility were not related to either EMF species richness or diversity. However, abiotic factors (MAT, MAP and soil C:N) did filter EMF community composition and the abundance of exploration types. As predicted, medium-fringe, and also contact explorers were more abundant in less fertile environments (as defined by lower pH, CEC, and available P), however, our second hypothesis was only partly confirmed because these exploration types were also associated with warmer or wetter environments. We did not find evidence for a functional connection between root diameter and EMF exploration type within Douglas-fir populations, which contradicts our third hypothesis.

### Ectomycorrhizal Fungal Richness and Diversity

Contrary to our first hypothesis, we found no evidence that EMF diversity and richness varied across environmental gradients. However, most of the studies that found temperature or soil fertility to have an impact on EMF diversity used experimental treatments or covered continental gradients (Deslippe et al., 2011; Suz et al., 2014; Haas et al., 2018; Köhler et al., 2018; Rosinger et al., 2018). It is then possible that results from our regional-scale biogeographic gradient may not directly compare to these studies with regard to EMF diversity. Nonetheless, EMF richness was not affected by climatic transfer in a genecological study in temperate rainforests of coastal British Columbia (Kranabetter et al., 2015b) or by experimental warming in boreal forests of Minnesota (Fernandez et al., 2017; Mucha et al., 2018).

In our study, EMF communities were dominated by host-generalist taxa such as *C. geophilum, L. rubrilactus* and *Russula*, whereas taxa in *Rhizopogon* and *Suillus lakei* that are specific to the Pinaceae and Douglas-fir, respectively, represented only 8% of the total colonized root tips. This pattern could explain the lack of changes in richness and diversity because the host-generalist taxa tend to be less sensitive to environmental changes (Bahram et al., 2012; Mucha et al., 2018). Alternatively, if rare or specialist taxa were to dominate EMF communities in our study, we could have observed a change in richness and diversity (Bahram et al., 2012; Mucha et al., 2018). It is important to mention that root tips communities in our study provide a measure of the investment of the host and fungus in nutrient exchange sites and enable assessment of fungal species abundance. However, it does not necessarily represent the community of extraradical hyphae, especially in the case of long-distance colonized root tips that have higher mycelial space occupation than medium-distance smooth and short-distance colonized root tips (Weigt et al., 2012). In addition, shifts in Douglas-fir rooting depth across regions may impact EMF diversity estimates (Supplementary Table 1; Pickles and Pither, 2014). Our sampling scheme was consistent along the gradient, which may have hindered the detection of changes in EMF richness and diversity deeper in the soil profile.

**Table 3:** Effect of fine-root (first-order) morphological and chemical traits on interior Douglas-fir ectomycorrhizal fungal species community composition across five regions assessed by PERMANOVA.

Fine-root traits	*Df*	*Pseudo F*	*Pseudo R^2^*	*P*-value
RTD^a^	1	1.88	0.03	**0.01**
Fine-root C:N^b^	1	1.93	0.03	**0.01**
Residuals	69		0.95	
Total	71		1.00	

*Significant P-value (<0.05) effects are shown in bold. ^a^RTD, root tissue density. ^b^Fine-root C:N, fine-root carbon-to-nitrogen ratio.*

### Abiotic Drivers at the Regional Scale

In our study, temperature, precipitation and soil C:N ratio moderately but significantly explained some of the changes in EMF community assembly, despite the relatively small ranges in temperature and soil fertility encompassed here. Hence, our first hypothesis was partly confirmed. We found that EMF communities varied from communities dominated by *Tomentella* and *Sebacina* in the colder, more fertile regions (higher soil pH, CEC and total N) of Douglas-fir’s natural range, to communities dominated by *Hydnum* sp., *Cortinarius* sp. or Russulaceae members in the warmer, less fertile regions of the range. These results add to those of Pickles et al. (2015a), who compared variation in EMF community primarily between inside and outside the range of Douglas-fir when studying EMF communities on Douglas-fir seedlings.

Our finding that temperature, precipitation and soil C:N ratio appeared to act as filters explaining part of the regional variation in EMF assemblages was similar to that of Pena et al. (2017). However, large-scale studies in Europe have shown different responses. For example, EMF community composition varied with temperature, pH and soil nutrients but not with precipitation in some European forests (Rosinger et al., 2018; van der Linde et al., 2018), whereas elsewhere precipitation, but not temperature, influenced EMF community structure (Jarvis et al., 2013; Suz et al., 2014).

In our study, the effect of temperature and soil fertility on EMF community structure could be related to co-evolutionary history between Douglas-fir populations and fungal symbionts (Gehring et al., 2017; Pither et al., 2018; Strullu-Derrien et al., 2018) because local adaptation of Douglas-fir populations is driven by temperature and soil N availability but can also be mediated by EMF (Rehfeldt et al., 2014; Kranabetter et al., 2015b; Pickles et al., 2015b). Temperature directly influences tree growth potential and may thus impact host C supply to fungal taxa. In turn this could induce a shift in EMF community structure across our study regions as EMF taxa differ in their C cost. Alternatively, temperature may have indirectly affected EMF assemblage through its impact on soil fertility such as availability of NO_3_^−^ and NH_4_^+^ (Kranabetter et al., 2015b). In addition to temperature, fitness and growth of Douglas-fir populations have been shown to relate to soil N availability (Kranabetter et al., 2015b), and close affiliation of Douglas-fir populations with local EMF symbionts may maximize tree nutritional adaptations (Kranabetter et al., 2015a; Leberecht et al., 2015). In turn this may reinforce the filtering effect of soil C:N ratio on EMF assemblage observed in our study.

### Taxonomic and Morphological Responses

We hypothesized that medium or long-distance explorers would be more abundant in colder climates or in soils with a high C:N ratio. Our results partly confirm this hypothesis as the hydrophobic, medium-fringe explorers *Cortinarius* sp., *Piloderma* sp., or *Amphinema* sp. and taxa in the Russulaceae classified as contact explorers, were more abundant in the warmer, less fertile environments of our study area, whereas the hydrophilic, short- and medium-distance smooth type, were more frequent and abundant in colder and more fertile conditions.

In our study system, this pattern of longer distance explorers associated with warmer climates can been linked to higher host photosynthetic capacity that can sustain more C demanding mycorrhizal symbionts (Jarvis et al., 2013; Fernandez et al., 2017; Köhler et al., 2018; Mucha et al., 2018; Rosinger et al., 2018). Furthermore, the positive response to temperature of the genera *Cortinarius* (except *C. decipiens*) and *Lactarius* are potentially related to the increased genetic capacity within these taxa for mobilization of N from organic matter (Bödeker et al., 2014; Kyaschenko et al., 2017). This may also hold true for the genus Russula as Jones et al. (2010) and Kyaschenko et al. (2017) highlighted the positive correlation between *Russula* taxa and enzymes mobilizing N and P from organic matter. Lilleskov et al. (2002, 2018) further classified *Cortinarius* and *Russula* as “nitrophobic” taxa. However, Looney et al. (2018) suggested that some members in the Russulaceae have lost the capacity to access C from organic matter.

The supposition that taxa associated with warmer climates tend to have competitive advantages in low N environments is supported by our data. Russulaceae and Cortinariaceae (with the exception of *C. decipiens*) were positively related to both C:N ratio and temperature. Consequently, in less fertile environments, fungi with proteolytic abilities such as *Cortinarius* or hydrophobic fungi with rhizomorphs such as *Piloderma* may be more competitive because they preferentially use insoluble, organic N. The latter fungi are likely less beneficial in richer soils (high total N concentration) where extensive exploration is not required (Ekblad et al., 2013; Koide et al., 2014; Suz et al., 2014). Similarly, Douglas-fir trees in the colder, more fertile environments of our study area may favor hydrophilic symbionts which potentially cost the plant less C, such as EMF with short emanating hyphae or “nitrophilic” EMF such as *Tomentella* (Nilsson and Wallander, 2003; Tedersoo and Smith, 2013; Haas et al., 2018).

Ascomycetes such as *Wilcoxina* sp., *Tuber* sp. and the drought-tolerant *C. geophilum*, exclusively occurred in the drier environments of our study area. This is in agreement with studies showing positive shifts in Ascomycetes abundance from mesic to xeric conditions (Allison and Treseder, 2008; Fernandez et al., 2017). It has been hypothesized that Ascomycetes have a lower C cost to their host due to their relatively thin mantles and contact or short-distance exploration types (Fernandez et al., 2017). This may be beneficial in the drier regions of southern British Columbia where water and carbon availability for growth is reduced and where lower basal area increment of Douglas-fir is accompanied by lower fine-root carbohydrate reserve concentration (Wiley et al., 2018). The long-distance explorer *Rhizopogon* also exclusively occurred in the drier climates of our study area. This likely represents host preference for drought-tolerant EMF as this taxon can transport water over long distances (e.g., Parke et al., 1983). As drier soils limit the diffusion rate of resources, this pattern of spatial niche separation could be an adaptation to stressful conditions (Pickles et al., 2015a). In addition, regions with drier soils were also phosphorus-limited, yet *C. geophilum* and *Rhizopogon*, both have a competitive advantage for plant nutrition in these conditions because the former possess acid phosphatase for P hydrolysis and mobilization, while the latter can forage for P more efficiently. This is because long-distance explorers have enhanced capacity for soil exploration and may therefore exploit soil resource such as P more completely (Kyaschenko et al., 2017; Köhler et al., 2018).

### Association Between Fine-Root and Mycorrhizal Traits

We expected fine-root diameter to be correlated with abundance of exploration types along the biogeographic gradient, yet we found RTD and fine-root C:N, but not diameter, to be significantly related to EMF community structure and patterns of exploration type frequency. Fine roots with lower tissue density occurred predominantly in colder regions (Defrenne et al., unpublished) and were more frequently uncolonized or colonized by EMF with short emanating hyphae. As we do not provide evidence for a functional connection between root diameter and mycorrhizal exploration types, EMF traits might not compensate for changes in fine-root structure.

Fungi with short emanating hyphae in colder conditions may instead serve a function to protect roots from environmental stresses (e.g., frost, pathogens; Marx, 1972). This would increase root persistence without investing as much in short hyphae construction and maintenance as in hyphae for long-distance exploration. In our study area, colder environments (excluding Revelstoke) were also poorer in available phosphorus, therefore, resistance to root pathogens, potentially conferred by short-distance type EMF, could be at the expense of efficient P exploitation, for which long-distance exploration types are thought to be better adapted (Köhler et al., 2018).

Alternatively, Zadworny et al. (2017) argue that lower RTD in absorptive roots could be due to increased percentage of mycorrhizal mantle area in the root, which would then relate to enhanced capacity for resource uptake. The cost of producing new root tips with low RTD is also lower than producing roots with high RTD, this potentially leads to increased efficiency in nutrient acquisition and thus to a more precise foraging strategy. In addition to contributing to the RTD, the mycorrhizal mantle can contribute to the chemistry of first- and second-order roots (Ouimette et al., 2013). For example, a significant proportion of the fine-root N of the Kamloops region (higher root N concentration) could be from fungal origin, particularly from the mantle formed by medium-fringe taxa such as *Cortinarius cedriolens* (Figure 2, 6) which colonized fine-root tips from Kamloops and had a slightly negative response to soil C:N (Figure 3).

In any case, selection for complementarity in foraging strategy was not a major mechanism within ectomycorrhizal tree species in a study by Chen et al. (2018a) but could be more common in arbuscular mycorrhizal tree species (Eissenstat et al., 2015; Liu et al., 2015; Zhang et al., 2019). Chen et al. (2018a) proposed bet hedging as a potential explanation because EMF traits selected for root pathogen protection may be at odds with those selected for resource foraging. Finally, the absence of a relationship between root diameter and exploration type abundance could be associated with the design of our study compared to that of Chen et al. (2018a). We used a regional scale biogeographic gradient and selected a ectomycorrhizal tree host that further expressed moderate intraspecific variation in root diameter compared to variation in RTD or root C:N, whereas Chen et al. (2018a) surveyed several ectomycorrhizal tree species with large differences in mean root diameter and investigated links between roots and ectomycorrhizal traits at the level of the nutrient patch.

## Conclusion

We combined fine-root and EMF trait measurements with next-generation sequencing across a biogeographic gradient. Douglas-fir EMF communities were dominated by host-generalist taxa which potentially explains the low variation in EMF α-diversity across environments. We did find, however, that temperature, precipitation and soil C:N ratio affected EMF community dissimilarities and exploration type abundance. Fungi with rhizomorphs (e.g., *Piloderma* sp.) or proteolytic abilities (e.g., *Cortinarius* sp.) dominated EMF communities in warmer and less fertile environments, whereas Ascomycetes (e.g., *C. geophilum*) or shorter distance explorers, which potentially cost the plant less C, were favored in colder/drier climates and richer soils (higher total N concentration). This pattern might be associated with co-evolutionary history between Douglas-fir populations and fungal symbionts, suggesting that the success of Douglas-fir as climate changes and stress increases may be dependent on maintaining strong associations with local communities of mycorrhizal fungi. At the regional scale, we did not find evidence for a functional connection between root diameter and EMF exploration types within Douglas-fir populations. Whether this implies no complementarity in resource foraging between fine roots and EMF is difficult to say, but this suggests that incorporating mycorrhizal symbiosis or at least EMF symbiosis into broader root trait frameworks may not be a suitable option if we are to represent the diversity of below-ground resource strategies. We thus encourage future research to simultaneously examine both root and fungal traits as separate entities.

## Data Availability

The datasets generated for this study are available on request to the corresponding author.

## Author Contributions

CD, WR, BP, and SS designed the study. CED wrote the manuscript. WR and CD collected the fine-root and mycorrhizal trait data. SG and CD carried out the molecular analyses. TP and CD carried out the data analyses. TP, BP, and SS contributed to the data interpretation, and drafted and edited the manuscript. All authors contributed critically to the drafts and gave final approval for publication.

## Conflict of Interest Statement

The authors declare that the research was conducted in the absence of any commercial or financial relationships that could be construed as a potential conflict of interest.

## Footnotes

1 http://forestrydev.org/biodiversity/bcern

2 www.deemy.de/
