# Supplementary material for: Shifts in Ectomycorrhizal Fungal Communities and Exploration Types Relate to the Environment and Fine-Root Traits Across Interior Douglas-Fir Forests of Western Canada
Source: Front Plant Sci. 2019 May 22;10:643. doi: 10.3389/fpls.2019.00643 (PMC6547044; doi:10.3389/fpls.2019.00643)
Supplement: Supplementary file 1 [file Data_Sheet_1.zip › Table 7.docx]

Supplementary Material

**Supplementary Figure 1.** Species accumulation curve using the Coleman method **(A)** and rarefaction curve **(B)** of ectomycorrhizal species richness identified from interior Douglas-fir root tips collected from 75 soil blocks across a biogeographic gradient. For reach region (Kamloops, Revelstoke, Salmon Arm, Williams Lake and Nelson), 15 soil blocks and 750 fine-root tips were extracted. The dotted lines correspond to the 95% confidence interval.

**(A)**


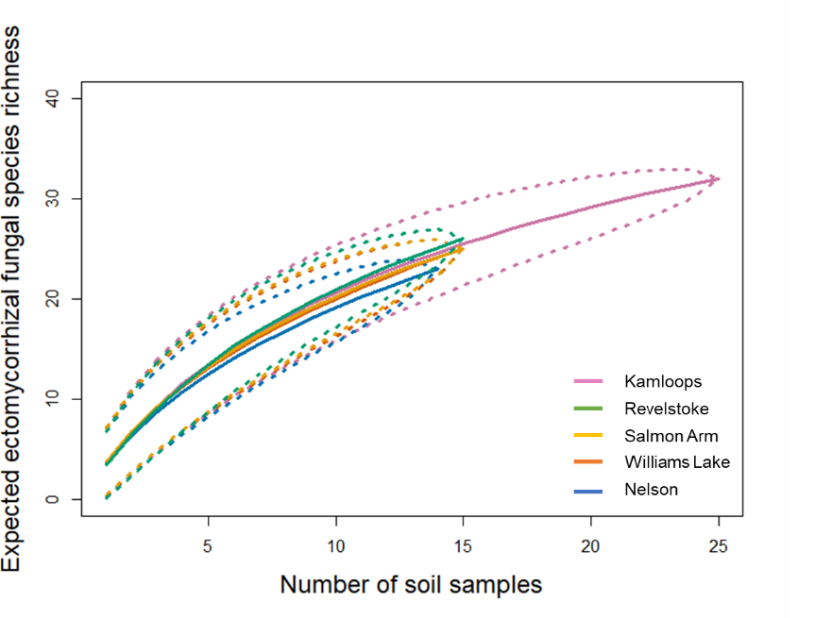


**(B)**

**
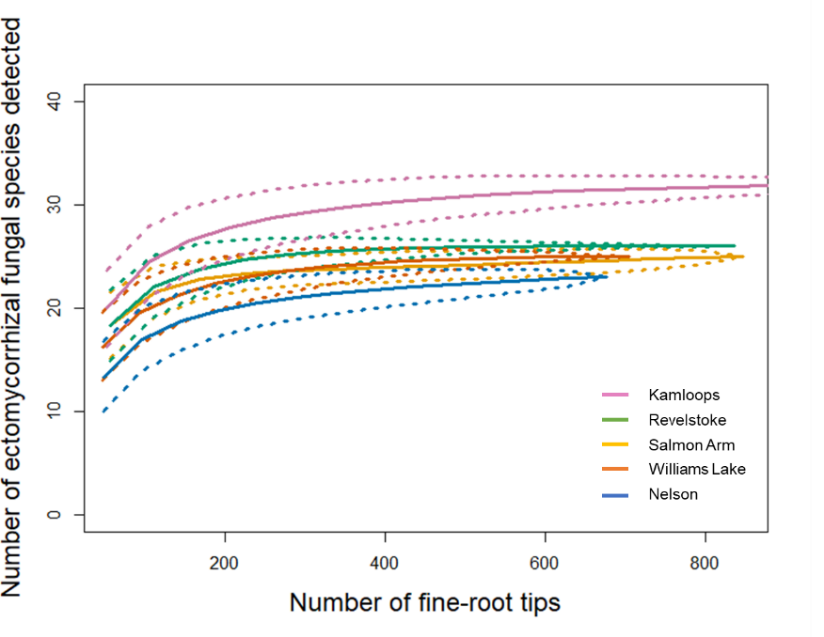
**

**Reference:**

Coleman, B.D., Mares, M.A., Willis, M.R., and Hsieh, Y. (1982). Randomness, area and species richness. *Ecology* 63: 1121–1133.

**Supplementary Figure 2.** Multivariate homogeneity of regions dispersion. The boxplot (**A**) shows the distance of values of β diversity of each region in relation to their centroids, calculated with the function ‘*betadisper*’ in the R package *vegan*. Non-euclidean distances between objects (**B**) and group centroids are handled by reducing the original distances to principal coordinates. R, Revelstoke; N, Nelson; SA, Salmon Arm; K, Kamloops; WL, Williams Lake.

**(A)**

**
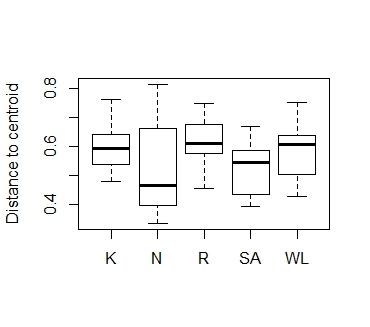
**

**(B)**

**
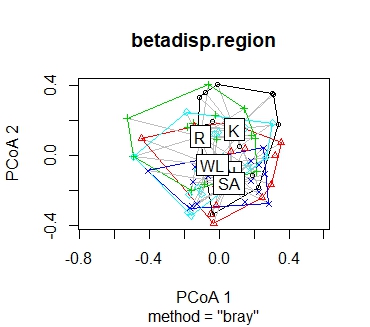
**

**Supplementary Figure 3.** Effect of environmental variables on Douglas-fir ectomycorrhizal fungal species community composition assessed by multivariate generalized linear models. On the figure, circles represent species coefficients and lines, 95% confidence intervals. This approach was used to account for the presence of mean-variance relationships in multivariate community analyses. Model significance was tested with likelihood-ratio test and univariate *P*-values were adjusted for multiple testing using a step-down resampling procedure.

MAT, mean annual temperature; MAP, mean annual precipitation; CNs, soil C:N ratio


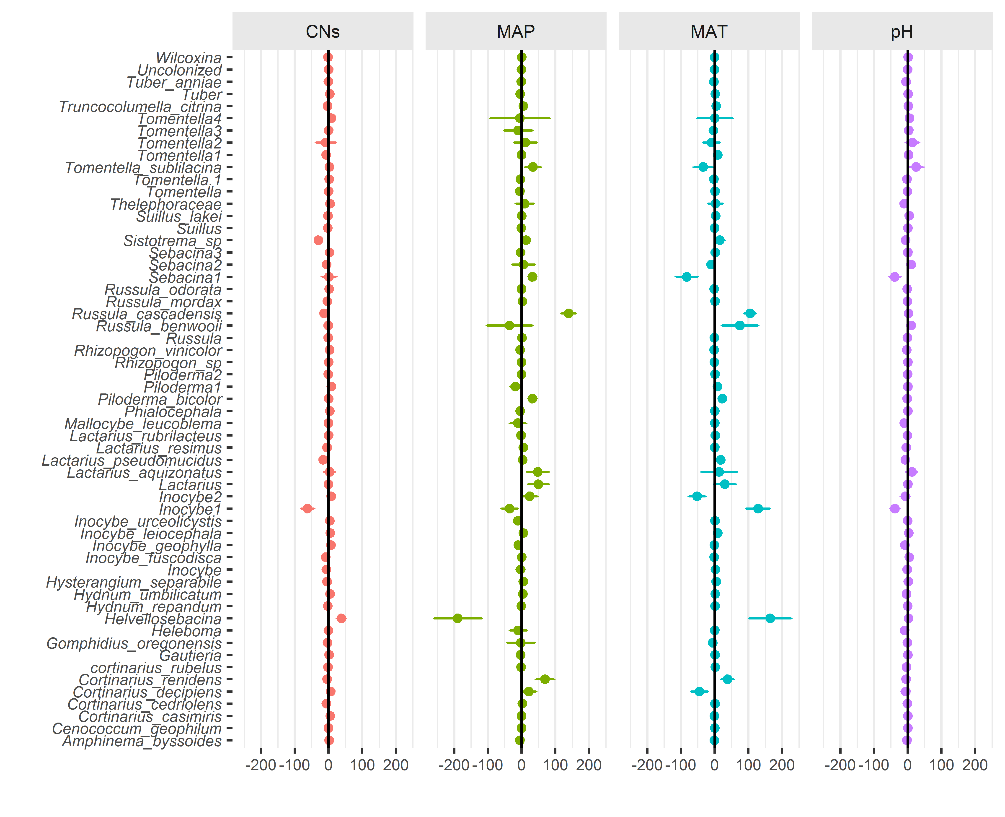


**Supplementary Figure 4.** Relative abundance of ectomycorrhizal fungal exploration types across a biogeographic gradient. Here, regions (Williams Lake, Revelstoke, Kamloops, Salmon Arm and Nelson) are classified from the coolest to the warmest region. For each species or genus, exploration types were assigned after Agerer (2001).

Supplementary Table 1**.** Stand properties of the 15 study stands selected across a biogeographic gradient in Western Canada. The soil texture was assessed on the first B horizon.

L, loam; SiC, silty clay; SiCL, silty clay loam; SiL, silt loam; SL, sandy loam

**Supplementary Table 2.** Description of morphological attributes of fine roots for three coniferous tree species encountered in this study. Description of root periderm texture and colour, root branching pattern and root tips habit are accompanied by exemplary pictures of root periderm and fine-root branching pattern. We validated the key with molecular genetic analysis of interior Douglas-fir (*Pseudotsuga menziesii* var *glauca*) and western hemlock (*Tsuga heterophylla* (Raf.) Sarg.; most similar morphologically). Samples were sent to the Appalachian laboratory at the Centre for Environmental Science (University of Maryland) and the findings of BLASTing Chloroplast DNA sequences from the rpl7 locus confirmed our expectations (accession numbers GQ999630.1, Douglas-fir and HQ846196.1, hemlock; Gugger et al.,2010).


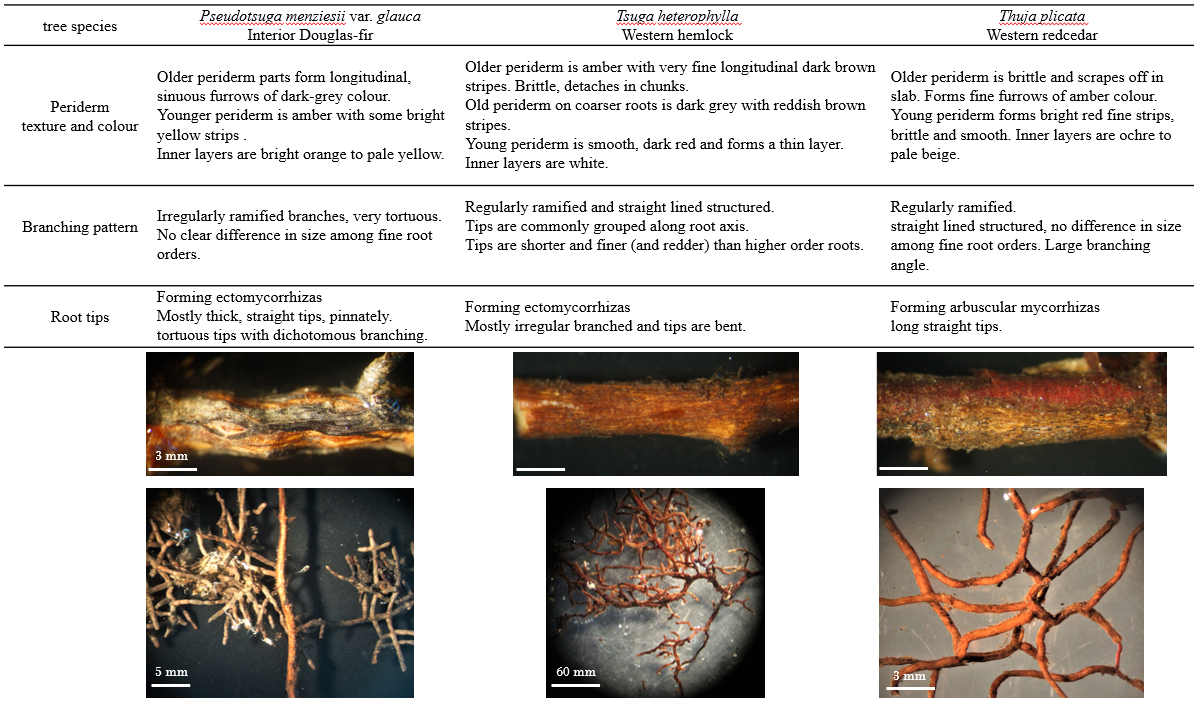


Reference: Gugger, P.F., Sugita, S., and Cavender-Bares, J. (2010). Phylogeography of Douglas-fir based on mitochondrial and chloroplast DNA sequences: testing hypotheses from the fossil record. Molecular Ecology *19*, 1877–1897.

**Supplementary Table 3**. See the file Supplementary_Table_3.xlsx

Operational taxonomic units (OTU) detected in each morphotype (in total 97 morphotypes). Each morphotype (1 excel sheet/morphotype) includes on average 70 OTUs (the rows). For each OTU, we report (i) the number of reads, (ii) the pairwise identity, (iii) the query coverage, (iv) the blast sequence matches and (v) the accession number in GenBank. Results are from BLAST GenBank without environmental sequences. For each morphotype, the OTUs highlighted in red are ectomycorrhizal OTUs and the OTU highlighted in green is the best BLAST sequence match. For further details, see the metadata excel sheet of the Supplementary Table 3.

**Supplementary Table 4**. Identity, relative frequency, exploration type and hydrophobicity of ectomycorrhizal fungi on interior Douglas-fir collected across an environmental gradient in western Canada. The relative frequency corresponds to the percentage of 3914 ectomycorrhizal root tips from 75 Douglas-fir trees in five regions that were colonised by the indicated species. Pairwise identity corresponds to the amount of nucleotide which matches exactly between two different sequences. For each species or genus, exploration types were assigned after Agerer (2001, 2006) and hydrophobicity was assigned based on Lilleskov et al. (2011) and Fernandez et al. (2017). ND, non determined; Ho, Hydrophobic; Hi, Hydrophilic.

**Supplementary Table 4**. **(continued)**

*** The morphotypes 32, 34b, 35 and 31 match the same best BLAST sequence (*Russula* sp.), however, they are likely two different species as they had very different morphologies.**

**References:**

Agerer, R. (2001). Exploration types of ectomycorrhizae. *Mycorrhiza* *11*, 107–114.

Agerer, R. (2006). Fungal relationships and structural identity of their ectomycorrhizae. Mycological Progress *5*, 67–107.

Fernandez, C.W., Nguyen, N.H., Stefanski, A., Han, Y., Hobbie, S.E., Montgomery, R.A. Reich, P.B., and Kennedy, P.G. (2017). Ectomycorrhizal fungal response to warming is linked to poor host performance at the boreal-temperate ecotone. Glob. Change Biol. 23, 1598–1609.

Lilleskov, E.A., Hobbie, E.A., and Horton, T.R. (2011). Conservation of ectomycorrhizal fungi: exploring the linkages between functional and taxonomic responses to anthropogenic N deposition. Fungal Ecology *4*, 174–183

**Supplementary Table 5.** Richness estimators and diversity indices per sample (**A**) of interior Douglas-fir ectomycorrhizal fungal species from five regions across a biogeographic gradient. Effect of region (**B**) on mycorrhizal richness, evenness and diversity assessed by nested ANOVA, region was a fixed effect and site nested within region, a random effect.

**(A)**

**(B)**

**Supplementary Table 6.** Effect of sites nested within regions **(A)** and regions **(B)** on Douglas-fir ectomycorrhizas. Hellinger transformation was applied to the species data matrix. The effect of sites within regions was assessed by PERMANOVA with permutations constrained within sites and the effect of region was evaluated with a nested analysis of variance. Significant *P*- values (<0.05) are shown in bold. This two-way approach was used to complement the db-RDA/PERMANOVA approach that were not developed to account for data with a nested structure (here, sites are nested within regions).

**(A)** Effect of sites within region

Model: ectomycorrhizal fungal species ~ Region/Site

Strata = Site

The residual error term give the correct test for the Region:Site interaction, but the test for the Region main effect is wrong because Site is the correct error term for testing it.

**(B)** Effect of regions

In this case, region is tested with site; site is tested with the residual error term but that latter test is not correct in this instance.

Supplementary Text 1. Soil sample analyses (**A**) and fine-root carbon and nitrogen concentration estimations (**B**).

**(A)** Soil sample analyses

Organic and mineral soil samples were air-dried and sieved to 2 mm. Samples were sent to the analytical laboratory of the B.C. Ministry of Environment (Victoria, British Columbia). Total soil C and N concentration (%) were measured using a combustion elemental analyser (Thermo Scientific Flash 2000 NC analyzer). For available phosphorus (PO_4_-P; orthophosphate as phosphorus), samples were prepared with the Bray P-1 method (dilute acid fluoride; Kalra and Maynard, 1991), and analysed with an UV/visible Spectrophotometer (Agilent Cary 60). To estimate the effective cation exchange capacity, cations were extracted from the soil samples with 0.1 M barium chloride (Hendershot and Duquette, 1986) and analysed with an ICP spectrometer (Teledyne Leeman, Prodigy Dual view).

(B) Fine-root carbon and nitrogen concentration estimations

To determine carbon and nitrogen concentration (%) in the first root orders, we randomly selected samples for each of the stands as follows; two soil blocks were selected out of five originally sampled per site, and two root branches were selected out of five originally sampled per block, for a total of 180 root samples (Thermo Scientific Flash 2000 NC analyzer).

**References:**

Hendershot, W.H., and Duquette, M. (1986). A Simple Barium Chloride Method for Determining Cation Exchange Capacity and Exchangeable Cations1. *Soil Science Society of America Journal*, *50*, 605. https://doi.org/10.2136/sssaj1986.03615995005000030013x

Kalra, Y.P., and Maynard, D.G. (1991). Methods Manual for Forest Soil and 552 Plant Analysis (125 p). Edmonton: Forestry Canada, Northwest region, Northern Forestry Center. Information Report NOR-X-319.
